# Indicators of the line of care for people with diabetes in Brazil: National Health Survey 2013 and 2019

**DOI:** 10.1590/SS2237-9622202200011.especial

**Published:** 2022-08-08

**Authors:** Deborah Carvalho Malta, Edmar Geraldo Ribeiro, Crizian Saar Gomes, Francielle Thalita Almeida Alves, Sheila Rizzato Stopa, Luciana Monteiro Vasconcelos Sardinha, Cimar Azeredo Pereira, Bruce Bartholow Duncan, Maria Inês Schimidt

**Affiliations:** 1 Universidade Federal de Minas Gerais, Escola de Enfermagem, Belo Horizonte, MG, Brazil; 2 Universidade Federal de Minas Gerais, Faculdade de Medicina, Belo Horizonte, MG, Brazil; 3 Ministério da Saúde, Departamento de Análise em Saúde e Vigilância de Doenças Não Transmissíveis, Brasília, DF, Brazil; 4 Instituto Brasileiro de Geografia e Estatística, Diretoria de Pesquisas, Rio de Janeiro, RJ, Brazil; 5 Universidade Federal do Rio Grande do Sul, Programa de Pós-graduação em Epidemiologia, Porto Alegre, RS, Brazil

**Keywords:** Adult, Non-communicable Diseases, Diabetes *Mellitus*, Epidemiology, Health Surveys

## Abstract

**Objective:**

To compare health care indicators for adults with medical diagnosis of diabetes *mellitus* (DM) in Brazil, in 2013 and 2019, and analyze the indicators for 2019 according to sociodemographic characteristics.

**Methods:**

Cross-sectional study using data from the 2013 and 2019 National Health Survey. Care indicators were evaluated in people with medical diagnosis of DM.

**Results:**

DM prevalence increased from 6.2% (2013) to 7.7% (2019). Between 2013 and 2019, there was an increase in the use of medications (from 80.2% to 88.8%) and of medical care (from 73.2% to 79.1%), a reduction in the use of Popular Pharmacy Program medications (from 57.4% to 51.5%) and in follow-up with the same physician (from 65.2% to 59.4%). In 2019, poorer indicators were observed for individuals who were male, younger, Black and Brown, and with lower education and income.

**Conclusion:**

Most indicators remained similar in the last five years, with differences according to sociodemographic characteristics in 2019.

**Study contributions**

**Main results**

The prevalence of diabetes *mellitus* (DM) increased from 2013 to 2019. Compared to 2013, in 2019 there was an increase in the use of medications and in medical care, and a reduction in the use of medications provided by the Popular Pharmacy Program and in follow-ups with the same physician.

**Implications for services**

Comparing and analyzing care indicators for DM according to sociodemographic characteristics can support the planning of actions for the control, prevention, treatment and evaluation processes in care pathways.

**Perspectives**

It is still necessary to support public policies for the monitoring of care management and care indicators, which point to the immense contribution of the Brazilian National Health System (SUS) in the pursuit of equity, comprehensiveness and reduction of health inequalities.

## Introduction

Diabetes *mellitus* (DM) is a non-communicable disease (NCD) related to increased blood glucose levels, which may result in repercussions on target organs such as the heart, blood vessels, the eyes, the kidneys and nerves.^1^

Worldwide, approximately 422 million people have DM and 1.6 million annual deaths are directly attributed to DM.^2^ According to data from the Global Burden of Disease (GBD), in 2019, DM was responsible for 2.74% (2.58%; 2.87%) of the total deaths in the world and 2.8% (2.5%; 3.1%) of years of life lost due to death or disability.^3^

Analysis of laboratory data from the 2013 National Health Survey (PNS) identified that the prevalence of DM can vary between 6.6% and 9.4% according to different criteria. In addition, it revealed that the prevalence of DM was higher for females, individuals aged over 30 years, with low education, overweight and obesity.^4^

The costs of DM are high and are associated with morbidity, mortality and complications, and may account for 15% of a country's annual health budget,^5^ considering direct expenses (medications, exams, procedures and supplies, professional visits and hospital expenses in emergency services, in addition to non-medical expenses) and indirect expenses (absenteeism from work, unproductivity, early retirement).^6^

For people with DM, access to treatment is essential for their survival. Aiming to strengthen health care for people with NCDs, including those with DM, the Ministry of Health published, in 2014, an ordinance that determines guidelines for the organization of lines of care for people with NCDs.^7^ Health care for the individual with DM, driven by clinical guidelines, aims to ensure health care, through monitoring and management, avoiding hospitalizations and deaths resulting from complications.^8^

The best evidence for DM management emphasizes the importance of structured lifestyle changes such as weight and blood sugar reduction, control of blood pressure, cholesterol, and multiple risk factors, as well as integrated, team-based, data-driven care.^9^

The monitoring of management and care indicators are important to assess DM care, allowing to uncover inequalities and, thus, support public policies. In Brazil, these indicators can be monitored through the PNS, which included questions about self-reported DM and about the care provided to this population. Thus, this study compared care indicators in adults with a medical diagnosis of DM in Brazil, between 2013 and 2019, and analyzed these indicators, in 2019, according to sociodemographic characteristics.

## Methods

This was a cross-sectional study that analyzed data from two editions of the PNS (2013 and 2019). The PNS is a household survey carried out by the Brazilian Institute of Geography and Statistics (IBGE) along with the Ministry of Health, aiming to produce data on lifestyles and the health status of the Brazilian population.

The PNS uses cluster sampling in three stages of selection, with the primary units being the census tracts, or a set of those, the secondary units being the households, and the tertiary units being the adult residents.^10^

In 2013, information was collected on 64,348 households, from approximately 80,000 selected households. In 2019, data were collected in 94,144 households, among the 108,525 selected.^10^ Details on the methodology can be found in specific publications.^10^

The following indicators, from 2013 and 2019, were compared:

- Prevalence of adults who have never measured their blood sugar level; and
- Prevalence of adults who reported a medical diagnosis of DM.

Individuals over 18 years of age, who reported a medical diagnosis of DM, were analyzed in the following indicators of DM care pathway:

- Used medication for DM or took insulin in the two weeks prior to the date of the interview;
- Received medical care for diabetes within the past 12 months;
- Last appointment for DM with the same physician as in previous appointments;
- Had all appointments with a specialist after referral;
- Had an eye exam within the past 12 months;
- Had a diabetic foot screening to check for sensitivity or the presence of wounds or rashes within the past 12 months;
- Hospitalization due to DM or some sort of complication;
- Severe or very severe degree of limitation in usual activities due to DM or some sort of complication;
- Last appointment for DM was at a Primary Health Care Center; and
- Obtained at least one medication through the “*Aqui Tem Farmácia Popular*” (Popular Pharmacy Program). More details on the construction of indicators are presented in Supplementary Material 1.

In 2019, care indicators were analyzed according to sociodemographic variables: sex (male; female); age group (18 to 29 years; 30 to 59 years; ≥ 60 years); self-reported race/skin color (White; Black; Brown - the other categories were added together, not being individualized due to the small number of observations); income [up to 1 minimum wage (MW); from 1 to 3 MWs; 3 or more MWs]; schooling (no schooling and incomplete elementary education; complete elementary education and incomplete high school; complete high school and incomplete higher education; complete higher education); and large regions (Midwest; North; Northeast; Southeast; South).

The 2013 and 2019 PNS database and questionnaires are available, for public access and use, in the PNS repository (https://www.pns.icict.fiocruz.br/).

Prevalence/proportions and 95% confidence intervals (95%CI) of the indicators were calculated for 2013 and 2019. Differences were evaluated using the chi-square test, considering p-value < 0.05

To calculate the prevalence ratios and 95%CI, Poisson regression models with robust variance were used, where the dependent variables were the indicators and the independent variables were the sociodemographic characteristics. The significance level adopted was 5%.

For data analysis, the Software for Statistics and Data Science (StataCorp LP, College Station, Texas, United States) version 14.0 was used, through the survey module, which considers the effects of the sampling plan.

In order to analyze the PNS data, it is necessary to define expansion factors or sample weights, with different probabilities of selection, both for households and for selected residents, due to the sample complexity. The result applied is a product of the inverse of the expressions of probability of selection of each stage of the sampling plan, which also includes correction for non-responses and adjustments of population totals.^10^ In order to ensure comparability between the two editions of the survey, IBGE carried out a new calibration of the expansion factors of the 2013 PNS considering the revision of the Population Projection of the Units of the Federation by sex and age.

Both studies were approved by the National Committee for Ethics in Research with Human Beings of the Ministry of Health, under No. 328,159 for the 2013 edition and No. 3,529,376 for the 2019 edition.

## Results

A total of 60,202 individuals were assessed in 2013, and 88,531 in 2019. Of these, 6.2%, in 2013, and 7.7%, in 2019 reported a medical diagnosis of DM and answered questions about care (Supplementary Material 2A). Furthermore, it was observed that, in 2013, 11.6% (95%CI 11.1;12.1) of the adult population had never measured their blood glucose, and in 2019, this prevalence dropped to 6.2% (95%CI 5.9;6.5) (Supplementary Material 2B).

**Figure 1:**
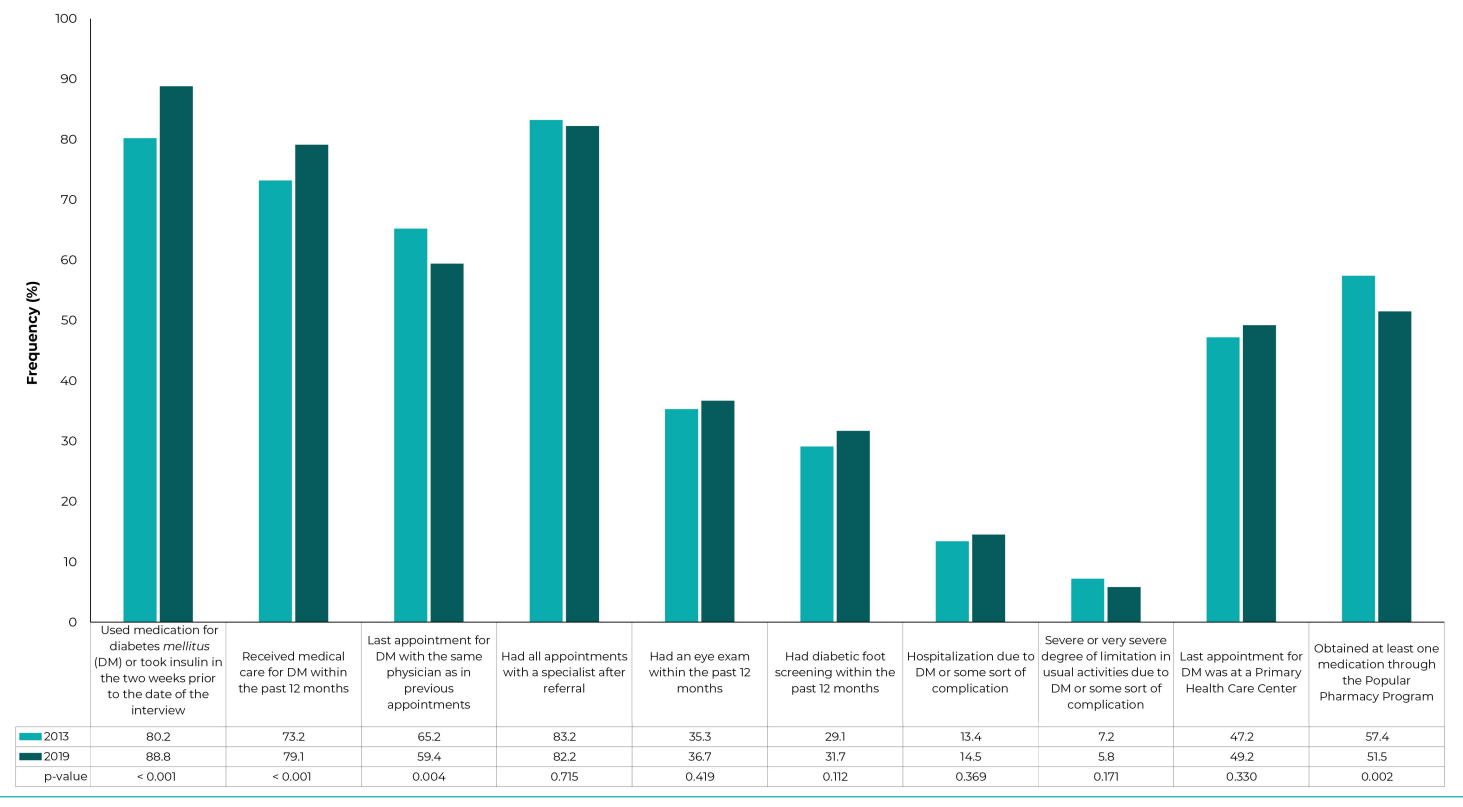
Health care indicators reported by Brazilians with diabetes *mellitus,* in the 2013 and 2019 National Health Surveys

Regarding health care, access and use of services among individuals with DM, there was an increase in the following: use of medication (from 80.2%, in 2013, to 88.8%, in 2019; p-value < 0.001) and the proportion of adults with DM who received medical care within the past year (from 73.2%, in 2013, to 79.1%, in 2019; p-value = 0.001). On the other hand, there was a reduction in medication obtainment through the Popular Pharmacy Program (from 57.4%, in 2013, to 51.5%, in 2019; p-value = 0.002), in the proportion of people with DM who reported having had an appointment with the same physician as in previous appointments (from 65.2%, in 2013, to 59.4%, in 2019; p-value = 0.004). No significant differences were found in the other indicators (Figure 1).

Analyzing the indicators according to sex, in 2019, it was observed that females: used the Popular Pharmacy Program more often to obtain medication (53.4%), had a higher proportion of medical assistance in the past year (81.0%), had their last appointment for DM follow-up at a PHC center (51.1%), and were hospitalized less often due to DM or complications (13.1%). The other indicators did not present significant differences (Table 1).

**Table 1:** Health care indicators reported by Brazilians with diabetes *mellitus* (n = 7,088), according to sex, with 95% confidence interval (95%CI), 2019 National Health Survey

Indicators	Sex		PR^a^ (95%CI)^b^
	Male (A)	Female (B)	
	% (95%CI)^b^	% (95%CI)^b^	B/A
Used medication for DM^c^ or took insulin in the two weeks prior to the date of the interview	88.8 (86.7;90.6)	88.9 (87.1;90.5)	1.00 (0.97;1.03)
Received medical care for diabetes within the past 12 months	76.6 (73.7;79.2)	81.0 (78.7;83.0)	1.06 (1.01;1.10)
Last appointment for DM with the same physician as in previous appointments	60.4 (57.1;63.6)	58.6 (55.7;61.5)	0.97 (0.90;1.04)
Had all appointments with a specialist after referral	82.7 (77.0;87.2)	81.9 (77.6;85.5)	0.99 (0.91;1.07)
Had an eye exam within the past 12 months	36.8 (33.8;39.8)	36.6 (34.1;39.3)	1.00 (0.90;1.11)
Had diabetic foot screening within the past 12 months	30.5 (27.8;33.4)	32.5 (30.1;35.1)	1.07 (0.95;1.20)
Hospitalization due to DM or some sort of complication	16.5 (14.3;19.1)	13.1 (11.5;15.0)	0.80 (0.65;0.97)
Severe or very severe degree of limitation in usual activities due to DM or some sort of complication	6.1 (4.7;7.9)	5.6 (4.2;7.6)	0.93 (0.62;1.38)
Last appointment for DM was at a Primary Health Care Center	46.4 (43.2;49.6)	51.1 (48.1;54.0)	1.10 (1.01;1.21)
Obtained at least one medication through the Popular Pharmacy Program	49.0 (45.9;52.2)	53.4 (50.6;56.1)	1.09 (1.01;1.18)

a) PR: Prevalence ratio; b) 95%CI: 95% confidence interval; c) DM: Diabetes *mellitus*.

In terms of regional differences, taking the North region as a reference, it was found that in the Southeast, the South and the Midwest there was a higher proportion of DM medication obtainment through the Popular Pharmacy Program (56.4%, 59.1%, and 56.4%, respectively). The Northeast and the Southeast regions had a lower proportion of last appointment at a PHC center (49.6% and 46.2%). The Southeast and the South regions had the highest percentage of access to the same physician as in the last appointment (61.8% and 64.7%). The Southeast had the highest percentage of eye exams performed in the past 12 months (40.8%) and the lowest number of hospitalizations due to DM or complications (12.1%). The other indicators did not show significant differences (Table 2).

**Table 2:** Health care indicators reported by Brazilians with diabetes *mellitus* (n = 7,088), according to region, with 95% confidence interval, 2019 National Health Survey

Indicators	Region					PR^a^ (95%CI)^b^			
	North (A)	Northeast (B)	Southeast (C)	South (D)	Midwest (E)	B/A	C/A	D/A	E/A
	% (95%CI)^b^	% (95%CI)^b^	% (95%CI)^b^	% (95%CI)^b^	% (95%CI)^b^				
Used medication for DM^c^ or took insulin in the two weeks prior to the date of the interview	86.8 (83.5;89.5)	90.2 (88.2;91.8)	88.8 (86.2;90.9)	87.0 (83.3;90.0)	90.1 (86.9;92.6)	1.04 (1.00;1.08)	1.02 (0.98;1.07)	1.00 (0.95;1.05)	1.04 (0.99;1.09)
Received medical care for diabetes within the past 12 months	82.3 (78.5;85.6)	78.4 (75.5;80.9)	80.7 (77.4;83.6)	74.6 (69.6;79.0)	78.5 (74.0;82.5)	0.95 (0.90;1.01)	0.98 (0.93;1.04)	0.91 (0.84;0.98)	0.95 (0.89;1.02)
Last appointment for DM with the same physician as in previous appointments	51.7 (46.3;57.0)	55.5 (52.4;58.7	61.8 (57.8;65.6)	64.7 (60.4;68.8)	51.6 (45.5;57.5)	1.08 (0.96;1.21)	1.20 (1.10;1.35)	1.25 (1.11;1.42)	1.00 (0.85;1.17)
Had all appointments with a specialist after referral	82.7 (75.4;88.2)	75.6 (69.6;80.7)	83.1 (77.3;87.6)	89.3 (83.8;93.1)	80.8 (71.2;87.7)	0.91 (0.82;1.02)	1.00 (0.91;1.11)	1.08 (0.98;1.19)	0.98 (0.86;1.11)
Had an eye exam within the past 12 months	34.2 (29.7;38.9)	32.2 (29.5;35.0)	40.8 (37.3;44.3)	32.1 (28.2;36.3)	36.3 (31.8;41.1)	0.94 (0.80;1.11)	1.20 (1.02;1.40)	0.94 (0.78;1.13)	1.06 (0.88;1.28)
Had diabetic foot screening within the past 12 months	29.9 (25.6;34.7)	29.4 (26.7;32.2)	34.0 (30.6;37.4)	30.5 (26.6;34.8)	28.4 (23.9;33.7)	0.98 (0.82;1.17)	1.13 (0.95;1.36)	1.02 (0.83;1.25)	0.95 (0.76;1.19)
Hospitalization due to DM or some sort of complication	17.4 (13.5;22.2)	17.2 (14.8;20.0)	12.1 (10.1;14.5)	15.9 (12.5;20.1)	16.6 (13.0;20.9)	0.99 (0.74;1.32)	0.70 (0.51;0.95)	0.92 (0.65;1.29)	0.95 (0.67;1.34)
Severe or very severe degree of limitation in usual activities due to DM or some sort of complication	5.6 (3.8;8.1)	5.9 (4.8;7.1)	6.0 (4.1;8.7)	5.5 (3.7;8.0)	5.7 (4.0;7.1)	1.05 (0.69;1.60)	1.07 (0.63;1.82)	0.98 (0.57;1.67)	1.02 (0.61;1.69)
Last appointment for DM was at a Primary Health Care Center	56.6 (51.2;61.8)	49.6 (46.4;52.8)	46.2 (42.3;50.0)	55.7 (50.7;60.5)	49.5 (43.7;55.3)	0.88 (0.78;0.98)	0.82 (0.72;0.93)	0.98 (0.87;1.12)	0.88 (0.75;1.02)
Obtained at least one medication through the Popular Pharmacy Program	35.6 (30.7;40.9)	39.5 (36.4;42.7)	56.4 (52.8;60.0)	59.1 (54.5;63.6)	56.4 (51.6;61.1)	1.10 (0.94;1.31)	1.59 (1.35;1.86)	1.66 (1.41;1.96)	1.58 (1.34;1.87)

a) PR: Prevalence ratio; b) 95%CI: 95% confidence interval; c) DM: Diabetes *mellitus*.

With respect to age groups, adults aged 30 to 59 years and the elderly (60 or over) showed the highest proportion of medication or insulin use (87.0% and 91.1%, respectively), of medical care for DM in the past year (79.9% and 79.2%), of severe/very severe limitation of activities due to DM or complications (7.0% and 5.2%), and a lower proportion of hospitalization (14.4% and 13.9%). The other indicators showed no significant differences (Table 3).

**Table 3:** Health care indicators reported by Brazilians with diabetes *mellitus* (n = 7,088), according to age groups, with 95% confidence interval, 2019 National Health Survey

Indicators	Age groups			PR^a^ (95%CI)^b^	
	18 to 29 (A)	30 to 59 (B)	≥ 60 (C)	B/A	C/A
	% (95%CI)^b^	% (95%CI)^b^	% (95%CI)^b^		
Used medication for DM^c^ or took insulin in the two weeks prior to the date of the interview	60.1 (41.8;76.0)	87.0 (84.6;89.0)	91.1 (89.5;92.5)	1.45 (1.07;1.95)	1.52 (1.13;2.03)
Received medical care for diabetes within the past 12 months	57.9 (39.8;74.1)	79.9 (77.1;82.6)	79.2 (77.0;81.2)	1.38 (1.02;1.87)	1.37 (1.01;1.86)
Last appointment for DM with the same physician as in previous appointments	58.3 (39.8;74.7)	57.9 (54.4;61.3)	60.5 (57.8;63.2)	0.99 (0.72;1.37)	1.04 (0.76;1.43)
Had all appointments with a specialist after referral	58.5 (22.6;87.1)	82.3 (77.5;86.2)	83.1 (78.6;86.8)	1.41 (0.73;2.71)	1.42 (0.74;2.74)
Had an eye exam within the past 12 months	30.5 (16.5;49.3)	34.9 (31.7;38.3)	38.2 (35.7;40.7)	1.15 (0.65;2.03)	1.25 (0.72;2.19)
Had diabetic foot screening within the past 12 months	17.8 (9.5;30.9)	26.3 (23.3;29.6)	36.1 (33.7;38.6)	1.48 (0.81;2.70)	2.03 (1.11;3.68)
Hospitalization due to DM or some sort of complication	43.3 (25.6;62.8)	14.4 (12.3;16.7)	13.9 (12.2;15.7)	0.33 (0.21;0.53)	0.32 (0.20;0.51)
Severe or very severe degree of limitation in usual activities due to DM or some sort of complication	0.4 (0.0;1.7)	7.0 (4.9;9.8)	5.2 (4.3;6.3)	17.20 (3.97;74.42)	12.84 (3.05;53.95)
Last appointment for DM was at a Primary Health Care Center	54.7 (36.4;71.9)	51.6 (48.1;55.1)	47.2 (44.5;49.9)	0.94 (0.67;1.33)	0.86 (0.61;1.22)
Obtained at least one medication through the Popular Pharmacy Program	36.5 (21.6;54.7)	53.2 (49.7;56.8)	50.7 (48.1;53.2)	1.46 (0.91;2.34)	1.39 (0.86;2.22)

a) PR: Prevalence ratio; b) 95%CI: 95% confidence interval; c) DM: Diabetes *mellitus.*

Compared to the population with lower levels of schooling, a better performance in several indicators was observed for individuals with higher education, such as a higher proportion of individuals who: had their feet screened within the past year (41.2%; 95%CI 35.1;47.4); had an eye examination within the past year (50.6%; 95%CI 44.5;56.6); had an appointment with a specialist (95.2%; 95%CI 91.5;97.3); had an appointment with the same physician as in previous appointments (69.5%; 95%CI 63.9;74.9); and had less severe/very severe limitations due to DM or complications (2.0%; 95%CI 0.9;4.2). Regarding the obtainment of medications through the Popular Pharmacy Program, lower rates were observed for the population with complete higher education (40.7%; 95%CI 35.1;46.4), and also for the last appointment at a PHC center (17.5 %; 95%CI 12.2;23.5). The other indicators showed no significant differences when analyzed from the perspective of schooling (Table 4).

**Table 4:** Health care indicators reported by Brazilians with diabetes *mellitus* (n = 7,088), according to education, with 95% confidence interval, 2019 National Health Survey

Indicators	Education				PR^a^ (95%CI)^b^		
	No schooling and incomplete elementary school (A)	Complete elementary school and incomplete high school (B)	Complete high school and incomplete higher education (C)	Complete higher education (D)	B/A	C/A	D/A
	% (95%CI)^b^	% (95%CI)^b^	% (95%CI)^b^	% (95%CI)^b^			
Used medication for DM^c^ or took insulin in the two weeks prior to the date of the interview	89.6 (87.9;91.0)	85.2 (79.2;90.2)	87.9 (84.5;90.7)	91.0 (87.1;93.8)	0.95 (0.88;1.02)	0.98 (0.95;1.02)	1.02 (0.98;1.06)
Received medical care for diabetes within the past 12 months	79.5 (77.2;81.6)	76.2 (69.6;81.7)	79.5 (75.5;82.9)	79.8 (75.1;83.8)	0.96 (0.89;1.04)	1.00 (0.95;1.05)	1.00 (0.94;1.07)
Last appointment for DM with the same physician as in previous appointments	57.0 (54.1;59.8)	61.7 (55.9;67.3)	60.1 (55.5;64.6)	69.5 (63.6;74.9)	1.08 (0.98;1.2)	1.06 (0.96;1.15)	1.22 (1.11;1.34)
Had all appointments with a specialist after referral	80.0 (75.0;84.0)	80.4 (71.7;86.9)	82.3 (75.2;88.3)	95.2 (91.5;97.3)	1.00 (0.9;1.12)	1.03 (0.93;1.14)	1.19 (1.11;1.27)
Had an eye exam within the past 12 months	32.2 (29.7;34.9)	39.1 (33.0;45.6)	41.5 (37.2;45.9)	50.6 (44.5;56.6)	1.21 (1.01;1.46)	1.29 (1.13;1.47)	1.57 (1.36;1.81)
Had diabetic foot screening within the past 12 months	29.0 (26.5;31.5)	33.5 (28.4;39.0)	34.0 (29.7;38.5)	41.2 (35.2;47.4)	1.16 (0.96;1.39)	1.17 (1.0;1.37)	1.42 (1.2;1.69)
Hospitalization due to DM or some sort of complication	15.9 (14.1;17.9)	12.5 (9.1;16.9)	13.3 (10.6;16.6)	11.3 (7.2;17.3)	0.78 (0.56;1.09)	0.84 (0.65;1.08)	0.71 (0.45;1.11)
Severe or very severe degree of limitation in usual activities due to DM or some sort of complication	7.3 (5.7;9.3)	4.0 (2.5;6.4)	4.5 (2.8;7.1)	2.0 (0.9;4.2)	0.55 (0.32;0.93)	0.61 (0.36;1.05)	0.27 (0.12;0.61)
Last appointment for DM was at a Primary Health Care Center	58.3 (55.5;61.0)	48.7 (42.8;54.7)	38.0 (33.4;42.8)	17.5 (12.9;23.5)	0.84 (0.73;0.95)	0.65 (0.57;0.74)	0.30 (0.22;0.41)
Obtained at least one medication through the Popular Pharmacy Program	52.3 (49.5;55.0)	59.8 (54.2;65.2)	50.1 (45.5;54.7)	40.7 (35.3;46.4)	1.15 (1.03;1.27)	0.96 (0.86;1.07)	0.78 (0.67;0.9)

a) PR: Prevalence ratio; b) 95%CI: 95% confidence interval; c) DM: Diabetes *mellitus.*

In the analysis according to race/skin color, it was found that the last medical appointment for DM follow-up at a PHC center was more frequent among Black and Brown individuals (55.7% and 54.8%, respectively). On the other hand, the proportion of individuals who had an appointment with the same physician as in the last appointment was lower (55.1% and 56.9%, respectively), as was the proportion for having had the feet examined within the past year (24.7% and 29.5%, respectively). Individuals of Brown race/skin color showed a lower proportion of eye exams during the appointment (33.8%). No significant differences were observed in the other indicators (Supplementary material 3).

With regard to income, the best indicators were observed for the population with an income of 1 to 3 MWs and more than 3 MWs, respectively, as follows: the physician who cared for them in the last appointment was the same as in the previous ones (60.9% and 72.2%); they were able to get an appointment with a specialist physician (87.6% and 91.6%); underwent eye examination within the past year (39.1% and 51.9%); had their feet examined within the past 12 months (33.3% and 43.9%); and there was a lower proportion of hospitalization (11.8% and 7.5%) and disability (4.3% and 2.6%). In addition, they had fewer medical appointments at a PHC center (45.0% and 13.3%). Individuals with an income of 3 MWs or more presented lower proportions of medication obtainment through the Popular Pharmacy Program (38.4%). The other indicators showed no differences when analyzed from the perspective of income (Supplementary material 4).

## Discussion

The study compared indicators related to the line of care for people with DM from the 2013 and 2019 editions of the PNS. There was an increase in the use of medication or insulin and of medical care within the past year, a reduction in medication obtainment through the Popular Pharmacy Program and in follow-ups with the same doctor. In 2019, in the analysis according to sociodemographic characteristics, worse indicators were observed for males, younger people, of Black and Brown race/skin color, with lower levels of education and income.

The blood glucose test is essential to diagnose DM and its monitoring in PHC has already been recommended.^11^ The temporal evolution points to progress in Brazil, between 2013 and 2019, with a reduction in the proportion of people who never had a blood test. However, the prevalence of the disease has increased, which may be a result of improved diagnosis, but essentially due to population ageing and increased obesity resulting from a sedentary lifestyle and unhealthy diets.^1^ ^,^ ^12^

The increase in self-reported DM occurred for both sexes, although the prevalence is higher among females. Analysis performed with laboratory data from the 2013 PNS and associated with reported use of medication, led to the conclusion that the prevalence was higher in females (9.7%; 95%CI 8.6;10.7), when compared to males (6.9%; 95%CI 5.9;7.9).^4^ Studies highlight that aspects such as gestational diabetes and hormonal changes in menopause can increase abdominal adiposity and justify the increase in DM among women.^13^ However, in countries such as Australia^14^ and England,^15^ using laboratory criteria, the data were different from the ones observed in Brazil, indicating higher prevalence of DM among men.

Worse care indicators were observed in terms of a reduction in follow-ups with the same doctor, which can interfere with the user’s longitudinal care, monitoring and follow-up. The continuity of care programmed in the therapeutic plan, designed for each user, is essential for the good evolution of the cases.^9^ ^,^ ^16^

Regarding the indicators of the line of care in 2019, the guidelines of the Ministry of Health for PHC recommend an annual medical appointment for the person with DM,^11^ which was achieved, according to the present study, in approximately 80% of the individuals with DM, with no variation according to education, income or race. Variation was observed for the young population, with less than 60% of annual check-ups, which can be attributed to shorter time since diagnosis and lower adherence to treatment.^17^ Also among this population, a higher proportion of females was cared for.

These indicators point to the importance of the Brazilian National Health System (SUS), the largest provider of care for people with DM. However, in relation to access to specialist physicians and exams, although it is also high, there were differences according to level of education and income. In addition, the study points out regional differences, with worse indicators in the North and Northeast regions in terms of access to medications through the Popular Pharmacy Program and to the same doctor as in the last appointment. These are the regions with the worst economic indicators and the greatest gaps in health care, due to the lack of physical infrastructure and health professionals.^18^ ^,^ ^19^

The PNS also revealed that a high proportion of people with DM used medication in the two weeks prior to the interview, regardless of gender, region, race/skin color, schooling and income, which attests the importance of SUS in providing and dispensing free medication in PHC, thus expanding access and reducing inequalities.^20^ It is worth highlighting that the PHC center offer supplies such as test strips for blood glucose and glycosuria, in addition to hypoglycemic drugs and insulin. Obtaining medications via the Popular Pharmacy Program was mentioned by half of the population, although there has been a reduction since 2013, which can be explained by successive cuts in funding for the program after 2017.^21^

Lower use of medication among younger adults (18 to 29 years old) can be explained by the lower severity of the disease, which may favor management through non-pharmacological measures, such as diet and physical activity.^11^ ^,^ ^22^ Greater use among the elderly (≥ 60 years) may be due to the greater severity and association with other cardiovascular risks.^23^

After 10 years of DM, loss of visual acuity due to retinopathy is common, therefore, it is essential to start follow-up at the time of diagnosis of type 2 DM (DM2) and, within five years, for type 1 DM (DM1), and the eye exam should be repeated yearly.^11^ ^,^ ^24^ Among the most frequent complications, the diabetic foot and its consequences, such as chronic wounds and infections, even lower limb amputations, stand out.^16^ Diabetic foot screening should be performed in all individuals with DM2 at the time of diagnosis. Among the indicators evaluated, these presented the worst performances, with only 1/3 of the population reporting having been examined, with a higher proportion among those with higher education and higher income. These results were far below those of other countries, such as the United States, where a survey in 2010 showed that about 70% of individuals with DM underwent an annual eye and foot examination.^17^

The higher frequency of hospitalizations among young people aged between 18 and 29 years old, in the present study, can be explained by the higher prevalence of DM1 in young adults, due to the acute symptoms of the disease and non-adaptation to the new care routine^24^ and with lower adherence to care practices.^17^ Self-care and the empowerment of users in their care should be increasingly sought by health teams, aiming at a better quality of life and the prevention of more serious outcomes.^11^ ^,^ ^25^

Hospitalizations were similar in terms of race/skin color and education and were less frequent in the population with higher income, suggesting the role of social determinants of health and their preponderance in the incidence, prevalence and evolution of DM.^26^ ^,^ ^27^

Among the limitations of the study is the self-reported prevalence, subject to reporting bias. However, this measure has provided valid estimates of DM prevalence,^4^ ^,^ ^28^ working as a proxy for population prevalence. Other limitations include the lack of distinction between the type of DM (DM1 or DM2) and the absence of evaluations regarding the difference in the care provided to the two different groups. It is worth highlighting that small changes were made to the questions which generated the indicators, between 2013 and 2019, which may have compromised the comparison between the two editions.

The study showed an increase in self-reported medical diagnosis of DM in the past five years. Regarding the care received, most care indicators remained similar in the period and the worst indicators were observed in populations with lower education and income, of Black and Brown race/skin color, males and among younger individuals. Worse indicators were observed in the North and Northeast regions, highlighting the regional differences and care gaps, which must be addressed. However, most indicators showed positive results and point to the immense contribution of the SUS in the pursuit for equity in health, and its role in generating comprehensiveness and reducing inequalities in health. Concern is raised about the constant attacks on the SUS, as well as budget cuts, especially with the approval of Constitutional Amendment No 95, in 2016, which among other measures reduced health resources, for 20 years, leading to profound changes in health care.^21^ ^,^ ^29^

## Figures and Tables

**Figure:**
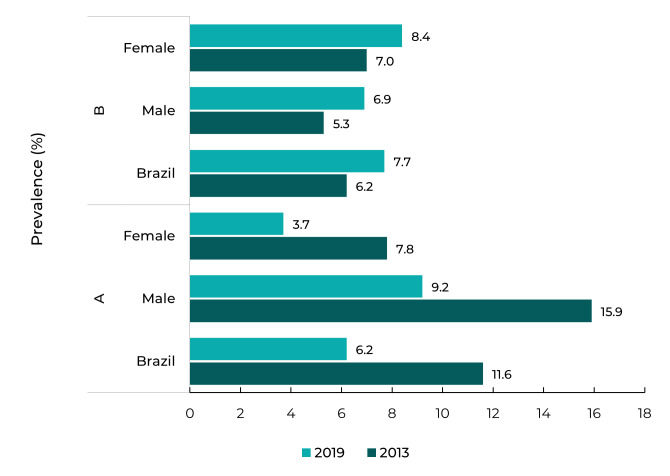
Prevalence of adults who have reported a medical diagnosis of diabetes *mellitus* (A), and of those who have never had a blood test to measure glycaemia (B), according to sex, National Health Survey, Brazil, 2013 and 2019

## Supplementary material 1

**Table:** Construction and calculation method of the indicators related to care and access to care for individuals with diabetes *mellitus*, National Health Survey, Brazil, 2013 and 2019

Indicator	Questions used in the construction of the indicator	Calculation method
Used medication for diabetes *mellitus* (DM) or took insulin in the two weeks prior to the date of the interview	**PNS 2013**	(number of individuals who have taken diabetes medication or insulin within the two weeks prior to the survey date/number of individuals who have reported a medical diagnosis of diabetes) x 100
	In the past two weeks, because of diabetes have you:	
	*Taken any oral medication to lower your blood sugar?* (yes; no)	
	*Taken insulin?* (yes; no)	
	**PNS 2019**	
	In the past two weeks, because of diabetes have you:	
	*Taken any oral medication to lower your blood sugar?* (yes; all of them; yes, some; no)	
	*Has any doctor ever prescribed insulin to you to control diabetes?* (yes; no)	
Received medical care for diabetes within the past 12 months	**PNS 2013 and 2019**	(number of individuals who have received medical care for diabetes within the past 12 months/number of individuals who have reported a medical diagnosis of diabetes) x 100
	*When was the last time you received medical care due to diabetes?* (less than 6 months ago; 6 months ago to less than 1 year; 1 year ago to less than 2 years; 2 years ago to less than 3 years; 3 years ago or over; never did)	
Last appointment for DM with the same physician as in previous appointments	**PNS 2013 and 2019**	(number of individuals who have reported that the doctor who they visited in the last appointment was the same as in previous appointments/number of individuals who have reported a medical diagnosis of diabetes and received medical care less than 3 years ago) x 100
	*In the last appointment, was the doctor who saw you the same as in previous consultations?* (yes; no)	
Had all appointments with a specialist after referral	**PNS 2013**	(number of people who have reported getting all appointments with a specialist doctor/number of individuals who have reported a medical diagnosis of diabetes and have been referred to an appointment with a specialist doctor) x 100
	*In any of the appointments for diabetes, was there a referral to a specialist doctor, such as a cardiologist, endocrinologist, nephrologist or ophthalmologist?* (yes; no; there was no referral, as all appointments for diabetes were with a specialist doctor)	
	*Did you go to all the appointments with the specialist doctor?* (yes; no)	
	**PNS 2019**	
	*In any of the appointments for diabetes, was there a referral to a specialist doctor, such as a cardiologist, endocrinologist, nephrologist or ophthalmologist?* (yes; no; there was no referral, as all appointments for diabetes were with a specialist doctor)	
	*Did you go to the appointments with the specialist doctor?* (yes, all of them; yes, some of them; no)	
Had an eye exam within the past 12 months	**PNS 2013 and 2019**	(number of individuals who have reported having an eye exam less than 1 year ago/number of individuals who have reported a medical diagnosis of diabetes) x 100
	*When was the last time you had an eye exam or ophthalmoscopy in which your pupil was dilated?* (less than 6 months ago; 6 months ago to less than 1 year; 1 year ago to less than 2 years; 2 years ago to less than 3 years; 3 years ago or over; never did)	
Had diabetic foot screening within the past 12 months	**PNS 2013 and 2019**	(number of individuals who have reported having had their feet examined less than 1 year ago/number of individuals who have reported a medical diagnosis of diabetes) x 100
	*When was the last time a doctor or healthcare professional examined your feet for sensitivity or the presence of wounds or irritations?* (less than 6 months ago; 6 months ago to less than 1 year; 1 year ago to less than 2 years; 2 years ago to less than 3 years; 3 years ago or over; never did)	
Hospitalization due to DM or some sort of complication	**PNS 2013 and 2019**	(number of individuals who have been hospitalized due to diabetes or some sort of complication/number of individuals who have reported a medical diagnosis of diabetes and received medical care less than 3 years ago) x 100
	*Have you ever been hospitalized because of diabetes or any complications?* (yes; no)	
Severe or very severe degree of limitation in usual activities due to DM or some sort of complication	**PNS 2013 and 2019**	(number of individuals who have reported severe/very severe degree of limitations in usual activities due to diabetes or some sort of complication/number of individuals who have reported a medical diagnosis of diabetes) x 100
	*In general, to what extent does diabetes or a diabetes complication limit your usual activities (such as working, doing housework, etc.)?*(does not limit; a little; moderately; severely; very severely)	
Last appointment for DM was at a Primary Health Care Center	**PNS 2013**	(number of individuals who have had their last appointment at a Primary Health Care Center/number of individuals who have reported a medical diagnosis of diabetes and received medical care less than 3 years ago) x 100
	*The last time you received medical care for diabetes, where did you receive care?* [PHC center (public health post or center or family health unit); specialty center, public polyclinic, or public medical assistance center (PAM); public emergency care center (UPA); another type of public emergency care center (24/7); public emergency room or the emergency department of a public hospital; public hospital/outpatient clinic; private doctor’s office or private clinic; Outpatient clinic provided by the company/business or the Union; private emergency room ou private hospital emergency department; At home, with a doctor from the family health team; At home, with a private practice doctor; other (specify)]	
	**PNS 2019**	
	*The last time you received medical care for diabetes, where were you seen?* [pharmacy; PHC center (public health post or center or family health unit); specialty center, public polyclinic, or public medical assistance center (PAM); public emergency care center (UPA); another type of public emergency care center (24/7); public emergency room or the emergency department of a public hospital; public hospital/outpatient clinic; private office, private clinic or private hospital outpatient clinic; private emergency room or private hospital emergency department; at home]	
Obtained at least one medication through the Popular Pharmacy Program	**PNS 2013**	(number of individuals who have obtained at least one medication or insulin via the Popular Pharmacy Program/number of individuals who have taken medication to control diabetes) x 100
	*Was any of the diabetes medications or insulin obtained from the Popular Pharmacy Program (PFP)?* (yes; all of them; yes, some; no)	
	**PNS 2019**	
	*Was any of the oral diabetes medications obtained from the “There is a Popular Pharmacy Here Program” (PFP) (“Aqui Tem Farmácia Popular”)?* (yes; all of them; yes, some; no)	
	*Was insulin obtained from the “There is a Popular Pharmacy Here Program” (PFP)?* (yes; no)	

## Supplementary material 3

**Table:** Indicators of care reported by Brazilians with diabetes *mellitus* (n = 7,088), according to self-reported race/skin color, with a 95% confidence interval, National Health Survey, Brazil, 2019

Indicators	Race/skin color			PR^a^ (95%CI)^b^	
	White (A)	Black (B)	Brown (C)	B/A	C/A
	% (95%CI)^b^	% (95%CI)^b^	% (95%CI)^b^		
Used medication for DM^c^ or took insulin in the two weeks prior to the date of the interview	88.3 (85.8;90.4)	88.8 (84.8;91.8)	89.1 (87.2;90.0)	1.00 (0.96;1.00)	1.00 (0.97;1.00)
Received medical care for diabetes within the past 12 months	77.8 (74.6;80.7)	77.9 (73.1;82.51)	80.5 (78.0;82.7)	1.00 (0.93;1.00)	1.00 (0.98;1.10)
Last appointment for DM with the same physician as in previous appointments	63.2 (59.6;66.6)	55.1 (49.0;61.3)	56.9 (53.7;60.0)	0.87 (0.77;0.98)	0.90 (0.83;0.97)
Had all appointments with a specialist after referral	85.1 (80.4;88.9)	83.2 (73.4;89.9)	78.3 (72.4;83.2)	0.97 (0.87;1.10)	0.92 (0.84;1.00)
Had an eye exam within the past 12 months	40.3 (37.3;43.45)	34.1 (29.1;39.4)	33.8 (30.9;36.9)	0.84 (0.71;1.00)	0.84 (0.74;0.94)
Had diabetic foot screening within the past 12 months	34.9 (32.0;38.0)	24.7 (20.7;29.2)	29.5 (26.7;32.5)	0.70 (0.58;0.85)	0.84 (0.74;0.96)
Hospitalization due to DM or some sort of complication	13.3 (11.2;15.6)	14.7 (11.5;18.7)	15.8 (13.8;18.1)	1.10 (0.82;1.49)	1.20 (0.96;1.40)
Severe or very severe degree of limitation in usual activities due to DM or some sort of complication	4.6 (3.5;6.0)	5.4 (3.9;7.4)	6.9 (4.9;9.5)	1.10 (0.77;1.80)	1.50 (0.96;2.30)
Last appointment for DM was at a Primary Health Care Center	41.5 (38.2;44.8)	55.7 (49.7;61.6)	54.8 (51.6;57.9)	1.30 (1.10;1.50)	1.30 (1.20;1.40)
Obtained at least one medication through the Popular Pharmacy Program	53.6 (50.5;56.6)	53.8 (48.0;59.5)	49.3 (46.1;52.5)	1.00 (0.89;1.10)	0.92 (0.84;1.00)

a) PR: Prevalence ratio; b) 95%CI: 95% confidence interval; c) DM: Diabetes *mellitus.*

## Supplementary material 4

**Table:** Indicators of care reported by Brazilians with diabetes *mellitus* (n = 7,088), according to income, with a 95% confidence interval, National Health Survey, Brazil, 2019

Indicators	Household income *per capita*			PR^a^ (95%CI)^b^	
	Up to 1 MW^d^ (A)	1 to 3 MWs^d^ (B)	3 MWs^d^ or more (C)	B/A	C/A
	% (95%CI)^b^	% (95%CI)^b^	% (95%CI)^b^		
Used medication for DM^c^ or took insulin in the two weeks prior to the date of the interview	88.6 (86.6;90.4)	89.0 (86.8;90.9)	89.2 (84.7;92.5)	1.00 (0.97;1.04)	1.01 (0.96;1.05)
Received medical care for diabetes within the past 12 months	79.2 (76.7;81.5)	79.2 (76.4;81.8)	78.6 (74.0;82.6)	1.00 (0.96;1.04)	0.99 (0.94;1.05)
Last appointment for DM with the same physician as in previous appointments	55.1 (52.1;58.0)	60.9 (57.2;64.4)	72.2 (67.0;76.9)	1.11 (1.02;1.19)	1.31 (1.20;1.43)
Had all appointments with a specialist after referral	75.7 (70.0;80.6)	87.6 (83.8;90.6)	91.6 (86.8;94.8)	1.16 (1.07;1.25)	1.21 (1.12;1.31)
Had an eye exam within the past 12 months	30.9 (28.3;33.6)	39.1 (35.8;42.5)	51.9 (46.6;57.1)	1.27 (1.12;1.43)	1.68 (1.47;1.92)
Had diabetic foot screening within the past 12 months	27.3 (24.9;29.8)	33.3 (30.0;36.7)	43.9 (38.5;49.5)	1.22 (1.07;1.40)	1.61 (1.38;1.88)
Hospitalization due to DM or some sort of complication	18.5 (16.3;20.9)	11.8 (9.9;13.9)	7.5 (5.4;10.5)	0.64 (0.51;0.79)	0.41 (0.29;0.58)
Severe or very severe degree of limitation in usual activities due to DM or some sort of complication	7.9 (6.4;9.7)	4.3 (2.6;7.1)	2.6 (1.4;4.8)	0.55 (0.32;0.94)	0.33 (0.17;0.63)
Last appointment for DM was at a Primary Health Care Center	61.3 (58.4;64.1)	45.0 (41.3;48.6)	13.3 (10.2;17.3)	0.73 (0.67;0.80)	0.22 (0.17;0.29)
Obtained at least one medication through the Popular Pharmacy Program	53.6 (50.6;56.5)	53.1 (49.6;56.7)	38.4 (33.5;43.6)	0.99 (0.91;1.08)	0.72 (0.62;0.83)

a) PR: Prevalence ratio; b) 95%CI: 95% confidence interval; c) DM: Diabetes *mellitus;* d) MW: Minimum wage.
